# A Dose-Ranging Study of Epinephrine Hydrofluroalkane Metered-Dose Inhaler (Primatene^®^ MIST) in Subjects with Intermittent or Mild-to-Moderate Persistent Asthma

**DOI:** 10.1089/jamp.2019.1558

**Published:** 2020-07-28

**Authors:** Edward M. Kerwin, Donald P. Tashkin, Thomas R. Murphy, George W. Bensch, Tony Marrs, Mary Z. Luo, Jack Y. Zhang

**Affiliations:** ^1^ Clinical Research Institute of Southern Oregon, Medford, Oregon.; ^2^ Division of Pulmonary and Critical Care Medicine, David Geffen School of Medicine, University of California at Los Angeles, Los Angeles, California.; ^3^ Charleston ENT and Allergy, Charleston, South Carolina.; ^4^ Allergy, Immunology, and Asthma Medical Group, Inc., Stockton, California.; ^5^ Amphastar Pharmaceuticals, Inc., Rancho Cucamonga, California.

**Keywords:** asthma, dose–response, efficacy, inhaled epinephrine, metered-dose inhalers

## Abstract

***Background:*** Two sequential single-dose crossover dose-ranging studies were performed to evaluate the clinical efficacy and safety profile of epinephrine hydrofluroalkane (HFA) metered-dose inhaler (MDI) formulation at various doses in subjects with asthma.

***Methods:*** In these multicenter, multiarm, double-blinded, or evaluator-blinded studies, subjects were randomized to receive the epinephrine HFA (Primatene^®^ MIST HFA) MDI medication at doses ranging from 90 to 440 μg/dose, as well as to a placebo (PLA) control and an active control of epinephrine CFC (chlorofluorocarbon) MDI (Primatene^®^ MIST CFC) at 220 μg/inhalation.

***Results:*** Spirometry testing for FEV1 (Forced Expiratory Volume in one second) demonstrated statistically significant improvements over PLA for epinephrine HFA MDI at all doses above 125 μg, as the amount out of the actuator (i.e., mouthpiece). The efficacy results for epinephrine HFA MDI in the dose range of 125–250 μg were also comparable to epinephrine CFC MDI (220 μg/inh). Safety assessments demonstrated minimal safety concerns for all treatment groups. No notable safety differences were observed between the studied doses of epinephrine HFA MDI and the active control formulation of epinephrine CFC MDI.

***Conclusion:*** The findings indicate that epinephrine HFA MDI provided clinically significant bronchodilator efficacy with minimal safety concerns in a dose range of 125–250 μg. These findings confirmed the optimal treatment doses of 125–250 μg that were appropriate for use in longer term 12 and 26 week chronic dosing studies of epinephrine HFA MDI for patients with intermittent or mild to moderate persistent asthma. Clinical trials registration number: NCT01025648.

## Introduction

An over the counter (OTC) epinephrine metered-dose inhaler (MDI), Primatene^®^ MIST CFC (220 μg/inh), was approved in the United States for the temporary relief of mild asthma for over 50 years (from 1956 to 2011). Due to the environmental concerns of CFC (chlorofluorocarbons), this product was phased out in December 2011 per the Montreal Protocol.^(1)^ Hydrofluroalkane (HFA) propellants have emerged as the best alternative to replace CFC propellants in MDIs leading to the development of a new epinephrine HFA inhaler. However, the development of a new formulation utilizing HFA propellant has proven to be a technical challenge since HFA tends not to function with CFC-compatible excipients due to differences in the chemical and physical properties of these two propellants.^(2–4)^ After much effort, a new epinephrine formulation utilizing HFA propellant was introduced and approved by the FDA on November 2018.

The new epinephrine HFA MDI, Primatene^®^ MIST, is the only FDA-approved OTC asthma medicine inhaler for the temporary relief of mild symptoms of intermittent asthma.^(5,6)^ Each spray delivers 0.125 mg of epinephrine (as the amount out of the actuator, i.e., mouthpiece). The new epinephrine MDI is approved for patients 12 years and older who have been diagnosed with intermittent asthma; and it should not be used more than 8 inhalations within 24 hours. Primatene^®^ MIST is not a replacement for long-term maintenance medications and patients are advised to visit a physician as part of both long-term and as-needed care.

Several key differences exist between the new epinephrine HFA and prior CFC version. First, the propellant was changed from CFC to HFA. More importantly, the physical property of the formulation was modified from a solution in the prior CFC version to a suspension. This allowed the epinephrine particles to be premicronized (size of <5 μm) in high amounts of HFA propellant. Owing to these changes, epinephrine HFA MDI was found to have a higher delivery efficiency.^(7)^ In detail, the aerosol characteristics of Epi-HFA and Epi-CFC, based on Anderson cascade analysis (6 sprays per test) are compared in Supplementary Appendix S1. As demonstrated in Supplementary Appendix S1, Epi-CFC has large amounts of epinephrine deposited before Stage-1 of the cascade (which will stop at the throat area of patients), whereas, Epi-HFA has large amounts of epinephrine deposited in Stages-3 to 5 of the cascade (this portion will reach the lungs of patients). Therefore, Epi-HFA has a higher delivery efficiency, which can potentially require a lower dose compared to the former CFC version to achieve therapeutic efficacy.

To determine the optimal dose of epinephrine HFA MDI, dose-ranging studies were completed. An optimal dose was targeted to be comparable if not better than the previously marketed epinephrine CFC MDI. This article presents findings from two sequential clinical dose-ranging studies (Trials A1 and A2) for epinephrine HFA MDI. Considering both studies jointly, the combined dose-ranging studies evaluated 90–440 μg/dose of epinephrine HFA MDI.

## Materials and Methods

### Study design

Two dose-ranging studies (A1 and A2) were conducted, in which various doses of epinephrine HFA MDI (Epi-HFA; Armstrong Pharmaceuticals, Inc., Massachusetts) were compared with the reference drug, epinephrine CFC MDI (Epi-CFC 1 × , or 2 × 220 μg; Armstrong Pharmaceuticals, Inc.), and placebo (PLA) in patients with intermittent or mild to moderate persistent asthma. The studies were performed at four to five study sites in the United States using a randomized, double-blinded or evaluator-blinded, single-dose crossover study design.

Initially, the study (study trial A1) tested five treatments, of which three doses of Epi-HFA at 250, 320, and 440 μg/dose (2 × 125, 2 × 160 and 2 × 220 μg) were compared to PLA (2 × PLA), and Epi-CFC 440 μg (2 × 220 μg). However, to provide a broader dose–response relationship and provide efficacy analysis at both single and dual inhalations of Epi-HFA, a second dose-ranging trial (study trial A2) of eight treatment arms was conducted. The study explored single and dual actuations of Epi-HFA at lower doses (1 × 90, 1 × 125, 2 × 90, 2 × 100, and 2 × 125 μg) compared to PLA (2 × PLA) and Epi-CFC (1 × 220, 2 × 220 μg).

Qualified subjects in the study were screened and enrolled based on fulfillment of the inclusion/exclusion criteria listed below. Each qualified subject participated in five or eight study treatment visits, which were separated by 2 to 14 days between treatment periods. At each study visit, subjects randomly received one of the following treatments per randomization code assigned: Epi-HFA (250, 320, or 440 μg), Epi-CFC (440 μg), or PLA-HFA in Trial A1, and Epi-HFA (90, 125, 180, 200, 250 μg), Epi-CFC (220 or 440 μg), or PLA-HFA in Trial A2. The Epi-HFA and PLA treatment inhalers had identical appearance and were double-blinded treatments. The Epi-CFC arms were evaluator-blinded due to their distinct product appearance.

Subjects were trained at screening and each treatment visit for correct dosing technique. Trained, qualified site staff technicians conducted the serial spirometry tests, and obtained laboratory and safety tests. The randomized inhalation treatments were self-administered, under the supervision of a designated unblinded dose-monitor, and in the absence of Pulmonary Function Technicians and investigators. FEV1 (Forced Expiratory Volume in one second) data were measured with a centrally dispensed spirometer to assess bronchodilator responses. At screening, FEV1 was measured pre- and postdose to determine the mean screening baseline FEV1 and reversibility. At subsequent treatment visits, FEV1 was measured at predose baseline, and serial FEV1 responses to inhaled study drugs were assessed at 5, 30, 60, 120, 180, 240, and 360 minutes postdose. After the final visit, each subject completed an end-of-study (EOS) safety evaluation.

The studies were conducted in accordance with the principles set forth in the ICH Good Clinical Practice guideline and the Declaration of Helsinki of the World Medical Association. The appropriate Institutional Review Boards/Ethics Committee approved the protocol and all study subjects provided written informed consent before initiation of the studies.

### Study subjects

Male and female adults aged 18–55 with intermittent or mild-to-moderate persistent asthma for at least 6 months, but who were otherwise generally healthy were screened. To qualify for the study, subjects were required to have a mean screening baseline FEV1 of 50–85% predicted normal per the National Health and Nutrition Examination Survey (NHANES) III reference values,^(8)^ and airway reversibility of ≥15% FEV1 within 30 (±5) minutes after inhaling 440 μg (2 × 220 actuations) of Epi-CFC at screening. Females could participate if they were nonpregnant and nonlactating and utilized a clinically acceptable form of birth control. Subjects had to demonstrate proficiency in the use of an MDI inhaler after training. Medical history was taken at screening. All subjects maintained their on-going prescribed or OTC asthma treatments after screening and during the study, but had to meet concomitant medication washout restrictions before each treatment visit as set forth in the protocol (Supplementary Appendix S2).

Subjects were excluded from the studies if they had life-threatening asthma, a smoking history of ≥10 pack-years, or smoking within 6 months before the screening. Asthma stability was ensured by excluding lower respiratory tract infections within 4 weeks and upper respiratory tract infections within 2 weeks before screening. Subjects were ineligible if they had current respiratory conditions other than asthma that might significantly affect the study or clinically significant concurrent illnesses. Subjects were also excluded if they had known intolerance or hypersensitivity to bronchodilator inhalers, had used prohibited drugs

(Supplementary Appendix S2), or had taken any Primatene^®^ MIST CFC within the last 30 days before screening.

### Efficacy variables

The primary efficacy endpoint was the area under the curve (AUC) of 0–6 hours, postdose percentage improvement in FEV1 compared with the predose baseline value (AUC_0–t_ of % ΔFEV1). The primary efficacy comparison was powered to show significantly greater bronchodilator efficacy observed with Epi-HFA doses compared to PLA.

The secondary efficacy variables include the following: AUC of postdose FEV1 volume change in liter-hours (AUC_0–t_ of ΔFEV1), peak bronchodilator effect or maximum change from baseline in FEV1 (maximum %ΔFEV1 or F_max_), time to peak FEV1 effect (t_max_), time to onset of bronchodilator effect (t_onset_) where %ΔFEV1 first reached a ≥12% increase from baseline, duration of bronchodilator effect of ≥12%, bronchodilator response rate (% responders) defined as the percentage of responders who demonstrated ≥12% improvement, and the % change in FEV1 at time points 5, 30, 120, 180, 240, and 360 minutes postdose. These endpoints evaluated the dose–response relationships of Epi-HFA compared to PLA and the active control medication Epi-CFC.

Spirometry was performed in conformance with the current standards from the American Thoracic Society (ATS)/European Respiratory Society (ERS).^(9)^ Reference values were derived from the NHANES III standard predicted normal database.^(8)^

### Safety variables

Vital signs (including systolic and diastolic blood pressure and heart rate) and a 12 lead ECG (electrocardiogram) (with QTc analysis) data were obtained at screening and at each study visit. Physical examination and laboratory tests, including CBC and serum chemistry, were evaluated at screening and EOS. All adverse events (AEs) were recorded and assessed.

### Statistical analysis

The primary efficacy analysis was performed using the evaluable crossover population or per protocol population, which was defined as subjects who received study treatment and had evaluable efficacy data (AUC) for each crossover treatment arm to which they were assigned. The primary efficacy evaluation was performed to examine whether Epi-HFA has a significantly greater bronchodilator effect compared to PLA in terms of AUC_0–t_ for %ΔFEV1. The study used one-sided paired *t*-tests with *α* = 0.05 for all Epi-HFA arms versus PLA.

Similar to the primary efficacy analysis, pairwise evaluations of the bronchodilator effect (AUC_0–t_ %ΔFEV1) were also performed as secondary efficacy analyses to compare Epi-HFA treatment versus Epi-CFC and Epi-CFC versus PLA. Other secondary efficacy variables, such as time to peak effect, onset of bronchodilator effect, were analyzed using a similar one-sided paired *t*-test as described for the primary endpoint when appropriate. For responder evaluations, a chi-square test was used at *α* = 0.05 comparing responder rates for Epi-HFA doses versus PLA, Epi-HFA doses versus Epi-CFC, and Epi-CFC doses versus PLA. The primary endpoint and secondary endpoints were calculated with 95% confidence intervals (CIs) and *p*-values.

Safety parameters were analyzed in the treated population. Vital signs, ECG readings, physical examination findings, and clinical laboratory results at EOS were compared with the baseline data. In addition, postdose vital signs and ECG readings for each study visit were evaluated. AEs were recorded at the level of the Medical Dictionary for Regulatory Activities (MedDRA) preferred term.

## Results

### Study demographic

A total of 56 subjects were enrolled in trials A1 (*n* = 26) and A2 (*n* = 30) at four and five clinical sites, respectively. Study participants were 55% (*n* = 31) female and 45% (*n* = 25) male and had a mean age of 34 to 35 with the majority being Caucasian. The mean FEV1 percent of predicted was about 68% in both studies A1 and A2, respectively. Since each individual subject was planned to participate in all treatment arms of the crossover trials A1 or A2, the demographic data for each treatment group were similar (Supplementary Appendix S3).

### Efficacy analysis

#### Dose–response (90–440 μg)

Epi-HFA at all single doses studied (90, 125, 180, 200, 250, 320, and 440 μg) demonstrated FEV1 improvements relative to the baseline. Table 1 summarizes data of the primary endpoint (AUC of %ΔFEV1), as well as for four secondary endpoints, AUC ofΔFEV1, maximal %FEV1, duration of FEV1 ≥ 12%, and % responders. The 95% CIs as well as the *p*-value ranges of a *t*-test versus PLA for each efficacy parameters are also listed in Table 1. The additional secondary endpoints, t_max_ and onset time, were similar for all active doses, and therefore were not analyzed for dose–response assessments.

**Table 1.** Dose–Response of Epinephrine Hydrofluroalkane Metered-Dose Inhaler Efficacy Endpoints

Studied drugs	Dose	Study arms		Dose-dependent endpoints: mean (95% confidence interval)				
	μg	Study A1	Study A2	AUC, %ΔFEV1 primary endpoint	AUC, ΔFEV	F_max_	Duration	Responder
		(n)	(n)	% × hours	L × hours	%	hours	%
PLA	0	P (21)	P (26)	23 (8.6–38)	0.6 (0.3–0.9)	10 (7.4–13)	0.82 (0.38–1.2)	32 (19–45)
Epinephrine HFA MDI	90		T1 (25)	37 (17–58)	0.9 (0.4–1.4)	14 (11–17)	1.6 (0.84–2.4)	64 (45–83)
	125		T2 (25)	73 (47–98)	1.7 (1.1–2.3)	20 (16–24)	3.1 (2.0–4.2)	76 (59–93)
	180		T3 (25)	98 (74–122)	2.2 (1.7–2.7)	24 (20–28)	4.2 (3.2–5.1)	88 (75–101)
	200		T4 (25)	76 (52–100)	1.8 (1.3–2.3)	21 (16–25)	3.1 (1.9–4.2)	80 (64–96)
	250	T1 (21)	T5 (24)	87 (67–106)	2 (1.6–2.5)	22 (19–26)	3.4 (2.6–4.2)	76 (63–88)
	320	T2 (20)		63 (34–92)	1.5 (0.7–2.3)	19 (15–24)	3.2 (2.1–4.2)	75 (56–94)
	440	T3 (21)		76 (54–98)	1.9 (1.3–2.5)	21 (17–25)	3.4 (2.3–4.6)	81 (64–98)
Epinephrine CFC MDI	220		A1 (25)	63 (40–86)	1.5 (1.0–2.0)	18 (14–22)	2.7 (1.6–3.8)	68 (50–86)
	440	A (21)	A2 (24)	60 (45–75)	1.4 (1.1–1.8)	18 (16–21)	2.6 (1.9–3.3)	76 (63–88)
*p*-values for Epi-HFA vs. PLA (except Study A2 Arm-T1)				≤0.0004	≤0.0008	≤0.0001	≤0.001	≤0.008
*p*-values for Epi-CFC vs. PLA				≤0.02	≤0.004	≤0.0009	≤0.002	≤0.008

AUC, area under the curve; CFC, chlorofluorocarbon; HFA, hydrofluroalkane; FEV1, Forced Expiratory Volume in one second; MDI, metered-dose inhaler; PLA, placebo.

**FIG. 1.**
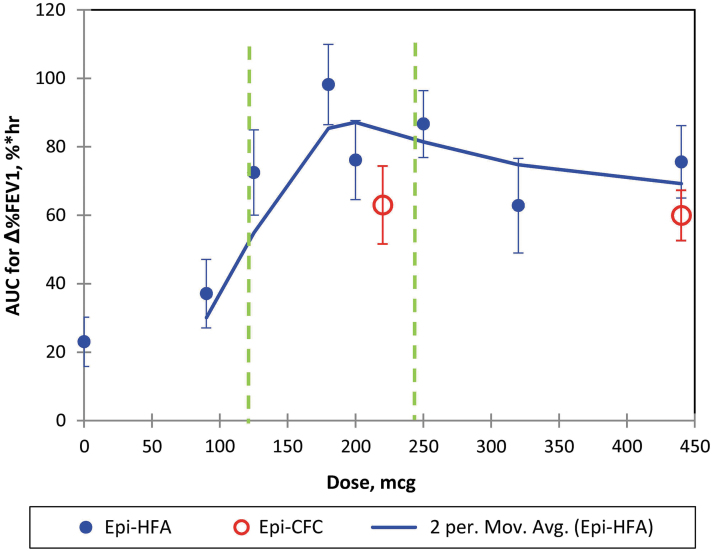
Primary endpoint of Epi-HFA versus Doses: AUC of %ΔFEV1: The two period moving average of Epi-HFA is shown with Standard Error Bars shown for each data point. The two green dash lines indicate the dose range that has been approved by the FDA. AUC, area under the curve; FEV1, Forced Expiratory Volume in one second; HFA, hydrofluroalkane.

The primary endpoint, AUC of %ΔFEV1, increased by 23% × hours with the pooled PLA doses, but showed a higher dose–response peaking in a range of 73–98% × hours AUC for doses in the range of 125–250 μg Epi-HFA (Table 1 and Fig. 1). The dose–response best-fit curve (Fig. 1) indicates all Epi-HFA doses, except the 90 μg dose, improved AUC of %ΔFEV1 compared to PLA, with a plateau and no further FEV1, increases observed above 250 μg.

**FIG. 2.**
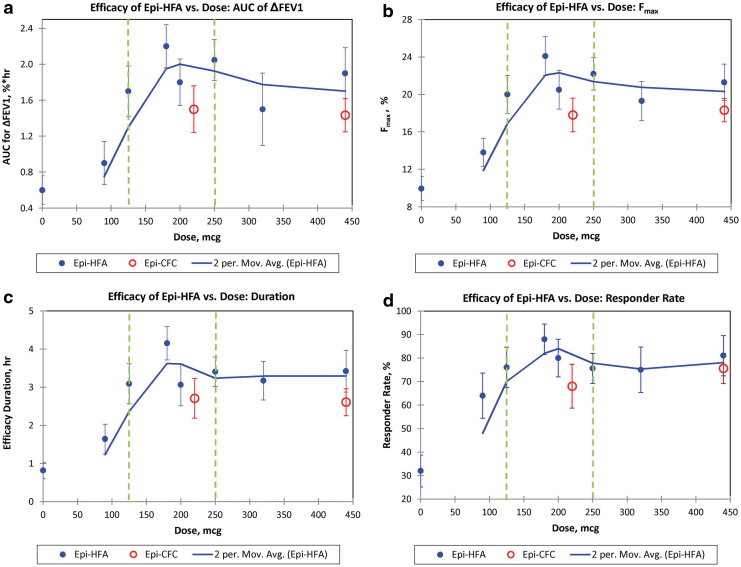
**(a–d)** Dose–response curves for four secondary endpoints. The two period moving average of Epi-HFA is shown with Standard Error Bars shown for each data point. The two green dash lines indicate the dose range that has been approved by the FDA.

A similar dose–response pattern was observed between active Epi-HFA doses for the four secondary dose-dependent endpoints (Fig. 2a–d). For these secondary variables, doses between 125 and 250 μg of Epi-HFA again provided highest responses in liter-hour improvements over 6 hours in FEV1 (AUC_0–t_ of ΔFEV1, Fig. 2a), and in maximal percent increase in %FEV1 (Fig. 2b), in duration of FEV1 increase exceeding ≥12% improvement (Fig. 2c), and in percentage of Responders with ≥12% increase in FEV1 (Fig. 2d). Therefore, these secondary endpoints further confirmed that doses between 125 and 250 μg appeared to give optimal bronchodilator benefit over PLA, and in a range comparable to the active control inhaler Epi-CFC.

In this study, the terms “comparable” and “similar” mean that Epi-HFA and Epi-CFC are clinically and statistically similar or comparable for efficacy evaluation. Table 2 shows *p*-values for the primary endpoint (AUC_0–6 hours_ of %ΔFEV1) for Epi-HFA and Epi-CFC versus PLA, which demonstrated efficacy in a broad dose range for both products (125 to 440 μg for Epi-HFA and 220 to 440 μg for Epi-CFC). The clinical results and statistical analysis for all secondary endpoints also show a similar efficacy profile. Therefore, Epi-HFA and Epi-CFC show clinically similar and statistically comparable efficacy.

**Table 2.** *p*-Values of Primary Endpoints for Epi-Hydrofluroalkane and Epi-CFC Versus Placebo

Studied drugs	Dose	Study and arms (*n*)		Mean and SD		p-values (vs.* PLA*)	Description on efficacy
	μg	Study A1	Study A2	AUC, %ΔFEV1			
		(n)	(n)	% × hours			
PLA	0	P (21)		22	41	—	—
	0		P (26)	24	56	—	—
Epinephrine HFA MDI	90		T1 (25)	37	50	0.1404	Not significant efficacy
	125		T2 (25)	73	62	<0.0001	Significant efficacy
	180		T3 (25)	98	59	<0.0002	Significant efficacy
	200		T4 (25)	76	58	<0.0003	Significant efficacy
	250	T1 (21)		89	70	<0.0001	Significant efficacy
	250		T5 (24)	85	63	<0.0001	Significant efficacy
	320	T2 (20)		63	62	0.0004	Significant efficacy
	440	T3 (21)		76	49	<0.0001	Significant efficacy
Epinephrine CFC MDI	220		A1 (25)	63	57	<0.0037	Significant efficacy
	440	A (21)		74	56	<0.0001	Significant efficacy
	440		A2 (24)	47	44	0.0196	Significant efficacy

CFC, chlorofluorocarbon; SD, standard deviation.

#### Details of trial A1: comparison of moderate doses (250, 320, and 440 μg)

**FIG. 3.**
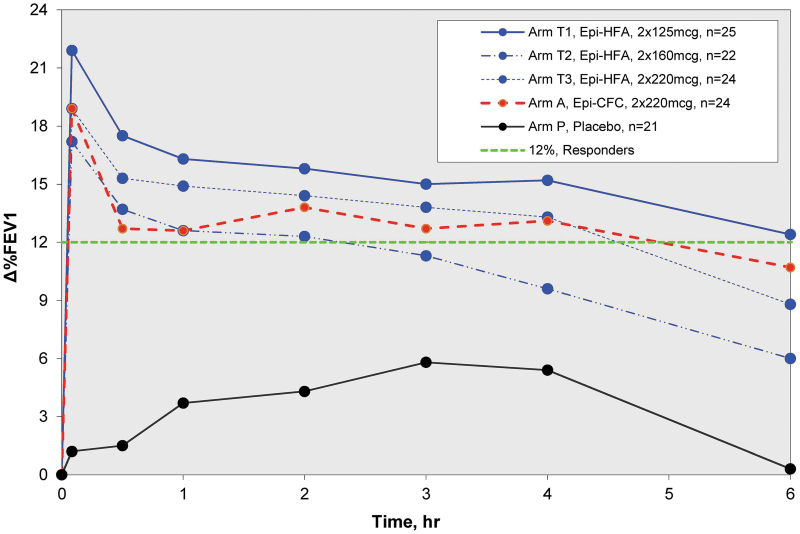
Zero to 6 hour spirometry results of Study A1: %ΔFEV1 Curves for Moderate Dose Study (250–440 μg/dose). All Epi-HFA doses tested in this study were well above PLA. The 2 × 125 μg showed greatest improvements in FEV1. PLA, placebo.

The initial dose-ranging trial A1 evaluated doses of Epi-HFA at 250, 320, and 440 μg, as well as PLA and the active control, Epi-CFC at 440 μg. Doses were given as two puffs of 125, 160, 220 μg of Epi-HFA, or 220 μg of Epi-CFC, respectively. Compared to PLA, all active doses of Epi-HFA and the Epi-CFC control group demonstrated significant bronchodilator efficacy results in % increase in FEV1 over 6 hours postdose (*p*-value ≤0.05) beginning 5 minutes postdose as indicated in Figure 3. The highest treatment response was seen with Epi-HFA 125 μg × 2 puffs, and this dose was comparable to the Epi-CFC 220 μg × 2 puffs. The efficacy profiles for all active doses provided comparable bronchodilation, with no statistically significant differences between active treatment arms. The %ΔFEV1 6-hours curve for Epi-HFA at 250 μg (2 puffs of 125 μg) was slightly higher than the 320, 440 μg, and Epi-CFC at 440 μg curves. No additional benefits for %ΔFEV1 were observed for Epi-HFA at total doses above 250 μg.

#### Details of trial A2: comparison of lower doses (90, 125, 180, 200, and 250 μg)

**FIG. 4.**
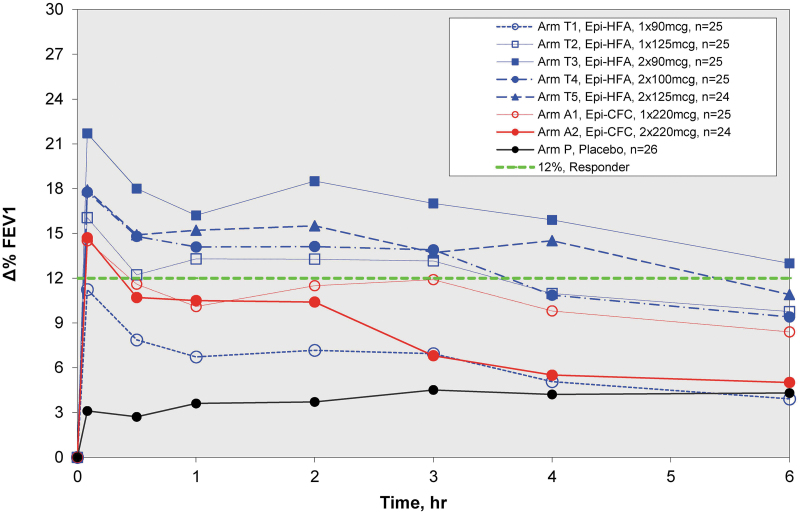
Zero to 6 hour spirometry results of Study A2. %ΔFEV1 Curves for Low-Dose Study (90–250 μg/dose). All Epi-HFA doses showed superiority to PLA, but the lowest Epi-HFA, 90 μg **(Arm T1)** showed minimal bronchodilation over PLA. 2 × 90 (180 μg) showed greatest improvements in FEV1.

A second crossover trial A2 was then conducted to look at lower doses of Epi-HFA. During eight crossover periods, Epi-HFA was evaluated at single puff doses, 90 and 125 μg, and dual puff doses totaling 180, 200, and 250 μg compared to PLA and Epi-CFC 220 μg (one puff) or 440 μg (two puffs) (Fig. 4). Again, FEV1 showed rapid improvement by 5 minutes postdose with improvement sustained over 6 hours for most doses of Epi-HFA and Epi-CFC. However, the Epi-HFA 90 μg dose did not provide significant efficacy over PLA for the primary and most of the secondary endpoints. Doses of 125 μg or greater did provide significant improvements for primary and most secondary endpoints (Table 1). The %ΔFEV1 curve for the 125 μg dose was closest to the single actuation of the active control arm, Epi-CFC (1 × 220 μg). The 180 μg (2 × 90 μg/inh) dose demonstrated the greatest degree of FEV1 improvement.

### Safety variables analysis

The safety findings were based on analysis of a total of 358 treatments administered to 56 subjects, a total of 220, 54, and 84 treatments with Epi-HFA, PLA, and Epi-CFC, respectively. Vital signs, ECG, and AE data were recorded for all subjects. No significant differences were observed between groups for vital signs and ECG data.

A total of 71 AEs were reported during the dose-ranging trials, including 48 (22%), 6 (11%), and 17 (20%) AEs for Epi-HFA, PLA, and Epi-CFC treatments, respectively. For the 71 AEs reported, all were nonserious and resolved without residual effects. Three severe events were reported in the Epi-HFA arms. Two of these were classified as “definitely not related to the study drug” by site investigators, while the third, feeling jittery, was classified as “likely related to the study drug.”

The three most common AEs reported with an incidence ≥3.0% in any treatment arm were headache (*n* = 11), dysgeusia (*n* = 10), and feeling jittery (*n* = 8). Among the 11 events of headache, 9 events occurred in Epi-HFA and 2 in Epi-CFC groups. For dysgeusia, 5, 1, and 4 events occurred in Epi-HFA, PLA, and Epi-CFC, respectively. Feeling jittery was reported in 7 events in Epi-HFA and one event in Epi-CFC (Supplementary Appendix S4). These three AEs occurred in a total of 29 AE reports and encompassed 41% ( = 29/71) of all AEs that occurred in the studies.

Blood routine and biochemistry data, including total proteins, sodium, potassium, chloride, glucose, calcium, CO_2_, BUN, creatinine, ALP, ALT, AST, bilirubin, RBC, hemoglobin, hematocrit, and WBC with differential were collected before and at the end of the study in study trials A1 and A2 as part of the clinical safety evaluations. All the laboratory data were obtained with regular standard methods. There were no clinically significant differences for these blood chemistry data before (at screening) and at the EOS for all trials.

## Discussion

### Dose selection

The optimal dose of Epi-HFA was selected based on the data from the dose-ranging clinical study trials, A1 and A2. The combined data (Fig. 1) and %FEV1 curves (Figs. 3 and 4) demonstrated significant bronchodilator efficacy at all doses of 125 μg or higher (the 90 μg dose did not demonstrate significant efficacy). Efficacy reached a stable range near 250 μg (2 × 125 μg/inh), with total doses greater than 250 μg showing no additional benefit. The results indicated that one or two actuations of 125 μg provided rapid and sustained FEV1 improvement comparable to Epi-CFC.

While the 125–250 μg doses demonstrated a high and consistent efficacy, the 180 μg (2 × 90 μg) dose was associated with the greatest degree of bronchodilation and was a possible dose consideration for subsequent study. However, Epi-HFA is intended to utilize the same dosing instructions as Epi-CFC (440 μg/inh), which advises consumers to start with one inhalation, and then to administer a second inhalation only if there is insufficient relief. Therefore, both single and dual inhalation doses may be therapeutic for patients. Since the 1 × 90 μg offered an inferior benefit, the 2 × 125 μg dose was deemed the best choice due to having an adequate response at one or two actuations. Such flexible dosing of Epi-HFA may encourage better management of asthma, given the possibility that bronchoconstriction may be relieved with first inhalation, with the second puff administered if needed. The 2 × 90 μg dose would not provide the same relief potential due to the lack of significant efficacy shown for the first actuation. Given that all Epi-HFA actuations were well tolerated and that both single and dual actuations were effective and comparable to Epi-CFC, the 125–250 μg range was determined to be the proper dose for Epi-HFA for subsequent clinical trials.

#### Comparison with active control (epinephrine CFC MDI)

Epi-HFA was compared with the active control, Epi-CFC in both dose-ranging trials. The %ΔFEV1 curves (Figs. 3 and 4) indicate that the Epi-HFA doses of 125–250 μg provide comparable or greater bronchodilator efficacy when compared to Epi-CFC (220–440 μg). With a 43% reduction in dose, the efficacy profile of the proposed dose of Epi-HFA, 2 × 125 μg/inh performed similarly to Epi-CFC (2 × 220 μg/inh).

These dose-ranging studies did have certain limitations. The numbers of subjects for these trials were small, with 21–25 subjects in each dosing group. Moreover, the studies investigated only single doses of one to two puffs of Epi-HFA and Epi-CFC, so future studies of 12 weeks or up to 26 weeks of chronic dosing were needed to evaluate the long-term efficacy and safety of chronic dosing.

Strengths of these studies included the careful crossover design, whereby each subject served as his or her own control. Both studies did include PLA treatments and active control arms with Epi-CFC inhalations to better define the bronchodilator efficacy response curves. These studies also selected subjects who were highly reversible to Epi-CFC (>15%), thereby likely enhancing the sensitivity of both A1 and A2 studies to determine the optimal bronchodilation doses versus Epi-CFC.

In conclusion, the two carefully performed single-dose crossover studies demonstrated that the 125 μg dose strength of epinephrine HFA MDI, one or two puffs, was significantly better than PLA and provided rapid and sustained bronchodilation up to 6 hours postdose comparable to the dose strength of epinephrine CFC MDI at 220–440 μg. All doses were safely tolerated, with the most common AEs reported to be headache, dysgeusia, and feeling jittery, which may represent class effects of inhaled adrenergic bronchodilators.

## Supplementary Material

Supplemental data

## Supplementary Material

Supplementary Appendix SA1

Supplementary Appendix SA2

Supplementary Appendix SA3

Supplementary Appendix SA4

## Notes

Reviewed by:

Tunde Otulana

Thomas O'Riordan

Gur Jai Pai Singh
