# Supplementary material for: A Dose-Ranging Study of Epinephrine Hydrofluroalkane Metered-Dose Inhaler (Primatene® MIST) in Subjects with Intermittent or Mild-to-Moderate Persistent Asthma
Source: J Aerosol Med Pulm Drug Deliv. 2020 Jul 28;33(4):186–93. doi: 10.1089/jamp.2019.1558 (PMC7407001; doi:10.1089/jamp.2019.1558)
Supplement: Supplemental data [file Supp_Data.pdf]

Supplementary Data

Supplementary Appendix S1. The Aerosol Characteristics of Epi-HFA and Epi-CFC

		Mass of fine particles <5 micrometers/6 sprays, μg											
Products	*Lot-#*	*Prior Stage 1*	*Stage 1*	*Stage 2*	*Stage 3*	*Stage 4*	*Stage 5*	*Stage 6*	*Stage 7*	*Stage F*	*Total*	*Per spray, μg*	*Recovery, %*
Epi-HFA	Lot-1	234	5	11	62	171	233	24	5	9	753	126	100
(125 μg/	Lot-2	271	6	12	65	170	223	25	5	11	788	131	105
spray)	Lot-3	247	7	13	65	163	213	17	4	9	738	123	98
Epi-CFC	Lot-1	1151	15	8	27	64	47	14	6	3	1335	222	101
(220 μg/	Lot-2	1130	14	7	26	64	45	11	5	3	1305	218	99
spray)	Lot-3	1216	13	9	31	58	42	12	6	25	1412	235	107

CFC, chlorofluorocarbon; HFA, hydrofluroalkane.

Supplementary Appendix S2. Prohibited Medications and Minimum Drug Washout

Part I. Examples of medications prohibited before screening and during the entire study (table 1 of 2)[a]

Classification	*Brand*	*Generic*	*Washout*
Oral and parenteral corticosteroids	Orasone, deltasone	Prednisone	4 weeks
	Decadron	Dexamethasone	
	Cortef	Hydrocortisone	
	Prelone, pediapred	Prednisolone	
	Delta-cortef	Prednisone	
	Aristocort, kenacort	Triamcinolone	
	Kenalog	Triamcinolone	
	Medrol	Methylprednisolone	
	Solu-medrol	Methylprednisolone	
Oral and parenteral β-agonists	Brethine, bricanyl	Terbutaline	1 week
	Alupent	Metaproterenol	
	Isuprel	Isoproterenol	
Monoamine oxidase-a inhibitor (MAOI A)	Marplan	Isocarboxazid	1 week
	Aurorix, manerix, Moclodura	Moclobemide, NE, 5HT	
	Nardil	Phenelzine	
	Parnate	Tranylcypromine	
Monoamine oxidase-B Inhibitor (MAOI B)	Deprenyl	Selegiline	1 week
	Azilect	Rosagiline	1 week
Anti-IgE	Xolair	Omalizumab	4 weeks
Theophyllines	All oral/systemic forms		1 week
Tricyclic antidepressants	Elavil	Amitriptyline	2 weeks
	Tofranil	Imipramine	
	Sinequan	Doxepin	
	Aventyl, pamelor	Nortriptyline	
	Norpramine	Desipramine	
	Surmontil	Trimipramine	
	Asendin	Amoxapine	
	Vivactil	Protriptyline	
	Ludiomil	Maprotiline	
	Anafranil	Clomipramine	

[a]The listed drugs are only examples of drug classes. Compliance for any specific nonlisted drug is subject to investigator discretion.

Part I. Examples of medications prohibited[1]
before screening and during the entire study (table 2 of 2)

Classification		*Brand*	*Generic/other name*	*Washout*
Beta- blockers	Nonselective beta-blockers	Levatol	Penbutolol	2 weeks
		Blocadron	Timolol	
		Betapace	Sotalol	
		Corgard	Nadolol	
		Inderal	Propranolol	
	Nonselective with ISA[2]	Cartrol	Carteolol	
		Viskin	Pindolol	
	Nonselective with α1-blocker	Normodyne or Trandate	Labetolol	
		Coreg	Carvedilol	
	Selective beta-1 blockers	Brevibloc	Esmolol	
		Zebeta	Bisoprolol	
		Lopressor; Tolprol XL	Metoprolol	
		Bystolic	Nebivolol	
		Tenormin	Atenolol	
	Selective beta-1 with ISA[2]	Kerlone	Betaxolol	
		Sectral	Acebutolol	
Inhaled anticholinergics[3]		Spiriva	Tiotropium	2 weeks
		Atrovent	Ipratropium	
Narcotic analgesics		Morphine-class	Opioids	Chronic use prohibited; 24-hours washout for occasional use
		Other narcotics	Per investigator discretion	
LABA[4]		Serevent	Salmeterol	≥72 hours washout before screening FEV1. Needs to switch to SABA during study
		Advair	Salmeterol/Flovent	
		Foradil	Formoterol	
		Symbicort	Budesonide/formoterol	
Antihistamines with anticholinergic actions		Atarax, Vistaril	Hydroxyzine	5 days

(1) The list only contains examples of drug classes. Compliance for specific, nonlisted drugs is subject to physician discretion.
(2) ISA, Intrinsic Sympathomimetic Activity.
(3) Use of systemic anticholinergics for nonasthmatic indications is generally allowed, but is subject to 24 hours washout. Topical ophthalmological anticholinergics are allowed.
(4) All LABA and LABA-containing medications are subject to 72 hours washout before screening FEV1 tests, and need to be switched to SABA MDI treatments, with concomitant inhaled corticosteroid if applicable, during the entire study.
FEV1, Forced Expiratory Volume in one second; LABA, long-acting inhaled β-agonists; MDI, metered-dose inhaler; SABA, short-acting β-agonist.

Part II. Medications allowed during the course of the study[a]
(Subject to withholding time before and during, spirometry tests at Screening Visit and Visit 1 through 5)

Name or class of medications	*Time withheld (hours)*
1) Inhaled SABA	8
2) Inhaled epinephrine (i.e., epinephrine CFC-MDI)	6
3) Inhaled, intranasal, or topical corticosteroids (See examples below)	6
4) Cromolyn and nedocromil (all mast cell stabilizers)	6
5) Antihistamines	
Long-acting	24
Short-acting (exceptions: Atarax [Hydroxyzine] is prohibited throughout Screening and study, as indicated in Part I of Appendix II)	12
6) Oral and topical decongestants	8
7) Leukotriene receptor antagonists	
Singulair	24
Accolate	24
Zyflo	24

[a]Stable regimens of chronic medications that may be continued throughout the study provided the sufficient washout is observed.

Examples of Inhaled Corticosteroids
(Allowed, but subject to withhold before spirometry on screening and clinical visits)

Inhalant corticosteroids	Vanceril, Beclovent	Beclomethasone	PO inhaler	Inhalation
	Azmacort	Triamcinolone	PO inhaler	Inhalation
	Aerobid, Aerobi-M	Flunisolide	PO inhaler	Inhalation
	Pulmicort	Budesonide	PO inhaler	Inhalation
	Flovent	Fluticasone	PO inhaler	Inhalation
	Alvesco, or Omnaris	Ciclesonide	PO inhaler	Inhalation
	Asmanex	Mometasone	PO inhaler	Inhalation

Supplementary Appendix S3. Demographic Baseline Characteristics of Subjects in Study Trials A1 and A2

	Study trial A1					*Study trial A2*							
	Epi-HFA			*Placebo*	*Active Control (Epi-CFC)*	*Epi-HFA*					*Placebo*	*Active Control (Epi-CFC)*	
Items	*250 μg* n = *26*	*320 μg* n = *25*	*440 μg* n = *24*	*0 μg* n = *24*	*440 μg* n = *25*	*90 μg* n = *29*	*125 μg* n = *29*	*180 μg* n = *29*	*200 μg* n = *29*	*250 μg* n = *29*	*0 μg* n = *30*	*220 μg* n = *30*	*440 μg* n = *29*
Age (years)													
Mean ± SD	34.7 ± 12.0	34.8 ± 12.2	34.2 ± 12.0	34.2 ± 12.0	34.8 ± 12.2	35.7 ± 11.0	35.7 ± 11.0	35.7 ± 11.0	35.7 ± 11.0	35.7 ± 11.0	35.3 ± 11.0	35.3 ± 11.0	35.7 ± 11.0
Range groups	18–55	18–55	18–55	18–55	18–55	18–55	18–55	18–55	18–55	18–55	18–55	18–55	18–55
<18	0 (0%)	0 (0%)	0 (0%)	0 (0%)	0 (0%)	0 (0%)	0 (0%)	0 (0%)	0 (0%)	0 (0%)	0 (0%)	0 (0%)	0 (0%)
18–40	17 (65%)	16 (64%)	16 (67%)	16 (67%)	16 (64%)	17 (58.6%)	17 (58.6%)	17 (58.6%)	17 (58.6%)	17 (58.6%)	18 (60%)	18 (60%)	17 (58.6%)
41–64	9 (35%)	9 (36%)	8 (33%)	8 (33%)	9 (36%)	12 (41.4%)	12 (41.4%)	12 (41.4%)	12 (41.4%)	12 (41.4%)	12 (40%)	12 (40%)	12 (41.4%)
65–75	0 (0%)	0 (0%)	0 (0%)	0 (0%)	0 (0%)	0 (0%)	0 (0%)	0 (0%)	0 (0%)	0 (0%)	0 (0%)	0 (0%)	0 (0%)
>75	0 (0%)	0 (0%)	0 (0%)	0 (0%)	0 (0%)	0 (0%)	0 (0%)	0 (0%)	0 (0%)	0 (0%)	0 (0%)	0 (0%)	0 (0%)
Gender													
Female	14 (53.8%)	13 (52.0%)	13 (54.2%)	13 (54.2%)	13 (52.0%)	16 (55.2%)	16 (55.2%)	16 (55.2%)	16 (55.2%)	16 (55.2%)	17 (56.7%)	17 (56.7%)	16 (55.2%)
Male	12 (46.2%)	12 (48.0%)	11 (45.8%)	11 (45.8%)	12 (48.0%)	13 (44.8%)	13 (44.8%)	13 (44.8%)	13 (44.8%)	13 (44.8%)	13 (43.3%)	13 (43.3%)	13 (44.8%)
Weight and height													
Weight, kg	79.2 ± 20.3	79.5 ± 20.7	79.8 ± 21.1	79.8 ± 21.1	79.5 ± 20.7	78.0 ± 16.0	78.0 ± 16.0	78.0 ± 16.0	78.0 ± 16.0	78.0 ± 16.0	77.7 ± 15.8	77.7 ± 15.8	78.0 ± 16.0
Height, cm	172.2 ± 9.9	172.1 ± 10.1	172.3 ± 10.2	172.3 ± 10.2	172.1 ± 10.1	169.8 ± 10.5	169.8 ± 10.5	169.8 ± 10.5	169.8 ± 10.5	169.8 ± 10.5	169.8 ± 10.3	169.8 ± 10.3	169.8 ± 10.5
Race													
Asian	1 (3.8%)	1 (4.0%)	1 (4.2%)	1 (4.2%)	1 (4.0%)	2 (6.9%)	2 (6.9%)	2 (6.9%)	2 (6.9%)	2 (6.9%)	2 (6.7%)	2 (6.7%)	2 (6.9%)
African American	4 (15.4%)	4 (16.0%)	4 (16.7%)	4 (16.7%)	4 (16.0%)	2 (6.9%)	2 (6.9%)	2 (6.9%)	2 (6.9%)	2 (6.9%)	3 (10.0%)	3 (10.0%)	2 (6.9%)
Caucasian	19 (73.1%)	18 (72.0%)	17 (70.8%)	17 (70.8%)	18 (72.0%)	24 (82.8%)	24 (82.8%)	24 (82.8%)	24 (82.8%)	24 (82.8%)	24 (80%)	24 (80%)	24 (82.8%)
Hispanic/Latino	0 (0.0%)	0 (0.0%)	0 (0.0%)	0 (0.0%)	0 (0.0%)	1 (3.4%)	1 (3.4%)	1 (3.4%)	1 (3.4%)	1 (3.4%)	1 (3.3%)	1 (3.3%)	1 (3.4%)
Others	2 (7.7%)	2 (8.0%)	2 (8.3%)	2 (8.3%)	2 (8.0%)	0 (0%)	0 (0%)	0 (0%)	0 (0%)	0 (0%)	0 (0%)	0 (0%)	0 (0%)
Screening FEV1 (L)	2.54	2.51	2.52	2.52	2.51	2.48	2.48	2.48	2.48	2.48	2.49	2.49	2.48
Screening FEV1 (%)	68.3	68.1	67.5	67.5	68.1	68.2	68.2	68.2	68.2	68.2	68.4	68.4	68.2
% Reversibility, mean ± SD	22.4 ± 5.4	22.5 ± 5.5	22.6 ± 5.6	22.6 ± 5.6	22.5 ± 5.5	21.7 ± 8.8	21.7 ± 8.8	21.7 ± 8.8	21.7 ± 8.8	21.7 ± 8.8	21.9 ± 8.7	21.9 ± 8.7	21.7 ± 8.8
ICS user, *n* (%)	15 (57.7%)	15 (60.0%)	15 (62.5%)	15 (62.5%)	15 (60.0%)	18 (62.1%)	18 (62.1%)	18 (62.1%)	18 (62.1%)	18 (62.1%)	18 (60.0%)	18 (60.0%)	18 (62.1%)
ICS nonuser, *n* (%)	11 (42.3%)	10 (40.0%)	9 (37.5%)	9 (37.5%)	10 (40.0%)	11 (37.9%)	11 (37.9%)	11 (37.9%)	11 (37.9%)	11 (37.9%)	12 (40.0%)	12 (40.0%)	11 (37.9%)

ICS, inhaled corticosteroids; SD, standard deviation.

Supplementary Appendix S4. Summary of Adverse Events in Single Normal Dose Studies A1 and A2

No.	Body system/ADE	*Studies*	*Trial A*					*Trial A2*								*Trials A+A2*						
			T (Epi-HFA)					*T (Epi-HFA)*						*A*[a]								
		Treatment and arm	*T1*	*T2*	*T3*	*P*[a]	*A*[a]	*T1*	*T2*	*T3*	*T4*	*T5*	*P*[a]	*A1*	*A2*	*T (Epi-HFA)*		*P*[a]		*A*[a]		*All*
		Dose (μg)	*250*	*320*	*440*	*0*	*440*	*90*	*125*	*180*	*200*	*250*	*0*	*220*	*440*	*90 ~ 440*		*0*		*220 ~ 440*		
		No. of Patients (treatments)	*26*	*25*	*24*	*24*	*25*	*29*	*29*	*29*	*29*	*29*	*30*	*30*	*29*	*220*		*54*		*84*		*358*
	Body as a whole	MedDRA code																				
1	Feeling jittery	10016338	1	0	1	0	0	1	1	1	1	1	0	0	1	7	**3.2%**	0	0.0%	1	1.2%	8
2	Headache	10019211	0	2	1	0	1	1	2	2	1	0	0	0	1	9	**4.1%**	0	0.0%	2	2.4%	11
	Cardiovascular system																					
3	Migraine	10027599	0	0	0	0	0	0	0	0	0	1	0	0	0	1	0.5%	0	0.0%	0	0.0%	1
	Digestive system																					
4	Burning sensation	10006784	0	0	0	0	0	1	1	1	1	1	1	1	1	5	2.3%	1	1.9%	2	2.4%	8
5	Dysgeusia	10013911	0	0	0	0	0	1	1	1	1	1	1	2	2	5	2.3%	1	1.9%	**4**	**4.8%**	10
6	Nausea	10028813	0	1	0	0	0	0	0	0	0	0	0	0	0	1	0.5%	0	0.0%	0	0.0%	1
7	Throat irritation	10043521	0	0	0	0	0	0	1	0	1	0	0	1	0	2	0.9%	0	0.0%	1	1.2%	3
8	Vomiting	10047700	0	0	0	0	0	0	0	0	1	0	0	0	0	1	0.5%	0	0.0%	0	0.0%	1
9	Paraesthesia oral	10057372	0	0	0	0	0	0	0	0	0	0	0	1	0	0	0.0%	0	0.0%	1	1.2%	1
	Musculoskeletal system																					
10	Muscle strain	10050031	0	0	0	0	0	0	0	0	0	0	1	0	0	0	0.0%	1	1.9%	0	0.0%	1
11	Musculoskeletal chest pain	10050819	1	0	0	0	0	0	0	0	0	0	0	0	0	1	0.5%	0	0.0%	0	0.0%	1
	Nervous system																					
12	Tremor	10044565	0	0	0	0	0	0	0	0	0	0	0	1	0	0	0.0%	0	0.0%	1	1.2%	1
	Respiratory system																					
13	Bronchospasm	10006482	0	0	0	0	0	0	0	0	0	0	1	0	0	0	0.0%	1	1.9%	0	0.0%	1
14	Cough	10011224	1	0	0	0	0	0	0	0	1	0	0	0	0	2	0.9%	0	0.0%	0	0.0%	2
15	Dysphonia	10013952	0	0	0	0	0	0	0	1	0	0	0	0	0	1	0.5%	0	0.0%	0	0.0%	1
16	Lower respiratory tract infection	10024968	0	0	0	0	0	0	1	0	0	0	0	0	0	1	0.5%	0	0.0%	0	0.0%	1
17	Nasopharyngitis	10028110	0	0	0	0	0	0	1	0	0	0	1	0	2	1	0.5%	1	1.9%	2	2.4%	4
18	Productive cough	10036790	0	0	0	0	0	1	1	1	0	0	0	1	0	3	1.4%	0	0.0%	1	1.2%	4
19	Respiratory tract irritation	10038731	0	0	0	0	0	1	1	1	0	0	0	1	0	3	1.4%	0	0.0%	1	1.2%	4
20	Upper respiratory tract infection	10046306	0	0	0	0	0	0	0	0	1	0	1	0	0	1	0.5%	1	1.9%	0	0.0%	2
	Urogenital system																					
21	Cystitis	10011781	1	0	0	0	0	0	0	0	0	0	0	0	0	1	0.5%	0	0.0%	0	0.0%	1
22	Menstruation irregular	10022992	0	0	0	0	0	0	0	0	0	1	0	0	0	1	0.5%	0	0.0%	0	0.0%	1
	Skin and appendages																					
23	Burns second degree	10006802	0	1	0	0	0	0	0	0	0	0	0	0	0	1	0.5%	0	0.0%	0	0.0%	1
24	Thermal burn	10053615	0	0	0	0	0	1	0	0	0	0	0	0	0	1	0.5%	0	0.0%	0	0.0%	1
25	Oral herpes	10067152	0	0	0	0	0	0	0	0	0	0	0	0	1	0	0.0%	0	0.0%	1	1.2%	1
Total ADE incidences for each treatment			4	4	2	0	1	7	10	8	8	5	6	8	8	48	22%	6	11%	17	20%	71
Total ADE incidences for drug				10		0	1			38			6	16		48	22%	6	11%	17	20%	71

Bold values have an ADE incidence ≥3%.
[a]Arms T: Epi-HFA; Arms A: Epi-CFC; Arm P: Placebo.
ADE, adverse drug event.
